# Testicular Sarcoidosis: An Underdiagnosed Manifestation That Poses Unique Challenges

**DOI:** 10.7759/cureus.25942

**Published:** 2022-06-14

**Authors:** Ujval S Choksi, Gurdeep Singh

**Affiliations:** 1 Family Medicine, Ascension - Our Lady of Lourdes Memorial Hospital, Binghamton, USA; 2 Internal Medicine/Endocrinology, Our Lady of Lourdes Memorial Hospital, Binghamton, USA

**Keywords:** lung nodules, hilar lymphadenopathy, testicular cancer, testicular lesion, sarcoidosis

## Abstract

Testicular involvement is a rarely encountered complication of sarcoidosis. There are unique challenges involved when sarcoidosis affects the genitourinary system. These include differentiating the findings from testicular cancer, which often results in invasive procedures such as orchiectomies. It can also be a cause of secondary infertility in men. Additionally, it may also be underdiagnosed. Here, we describe a case where a patient showed a constellation of findings suggestive of sarcoidosis, along with testicular involvement at initial presentation. In this case, the diagnosis was made clinically with supporting laboratory, pathology, and ultrasound findings. The testicular findings were not biopsied, as the patient had easily accessible skin findings to confirm sarcoidosis. His testicular findings are continued to be monitored via ultrasound.

## Introduction

Sarcoidosis is a systemic disease prevalent globally that forms noncaseating granulomas, affecting mainly young adults [1]. It can affect any organ with diverse manifestations, and its clinical course varies from completely benign to significantly reducing the patient’s lifespan or quality of life.

In the United States, African Americans have a 2.4% lifetime risk of sarcoidosis, and it preferentially also afflicts patients who are Scandinavian, Irish, German, and West Indians [2]. Sarcoidosis is thought to be polygenic, occurring in genetically predisposed patients who come into contact with an environmental trigger [2].

The pathogenesis involves the activation of T-cells after an interaction between antigen-presenting cells and major histocompatibility complex-II. This sets off a cascade that results in granuloma production, which is non-necrotizing in most cases [1]. Sarcoidosis generally involves the lungs and thoracic lymph nodes, but it can also affect the eyes (uveitis), cutaneous tissue, cardiovascular system, upper respiratory tract, the nervous system, and other organs including the liver and spleen [3]. Due to the variation in presentation, there are no standardized diagnostic criteria for sarcoidosis. However, clinical presentation, imaging, histologic evidence of non-necrotizing granulomas, and exclusion of other granulomatous diseases are generally acceptable approaches to making the diagnosis [4].

However, at times, sarcoidosis can affect other organs too, including the genitourinary (GU) system [5]. This is a rare manifestation, as it encompasses 0.2% of cases of sarcoidosis. As of 2019, only 68 cases of GU sarcoidosis had been reported in the literature [6,7]. In these cases, the patients had a testicular mass, which was biopsied and verified to be sarcoidosis [5]. However, there is also an association between the overall incidence of sarcoidosis and testicular cancer, with the incidence of sarcoidosis being 100-fold higher in patients with known testicular cancer [6]. Due to this, patients with sarcoidosis who have a testicular mass pose a unique challenge in determining the etiology of the mass, whether it is from testicular sarcoidosis versus testicular cancer [6].

We are reporting a rare case of sarcoidosis with non-painful testicular involvement. The patient discovered multiple masses by self-exam and presented with a constellation of other symptoms and findings suggestive of sarcoidosis.

## Case presentation

A 40-year-old African American male with a past medical history of type II diabetes on insulin, gastroesophageal reflux disease (GERD), and a BMI of 41 presented with a concern to remove multiple raised, nontender plaques on his anterior shins. He had no surgical history, and his social history was positive for significant alcohol use, and daily cannabis use. He underwent corticosteroid injections of his skin findings, thought to be keloids at the time. Labs were subsequently drawn, which showed mild hypercalcemia of 10.8 (8.4-10.4 mg/dL). Due to chronic bilateral lower extremity edema, an echocardiogram was also ordered, which showed an ejection fraction of 60-65%, with no concerning findings.

**Figure 1:**
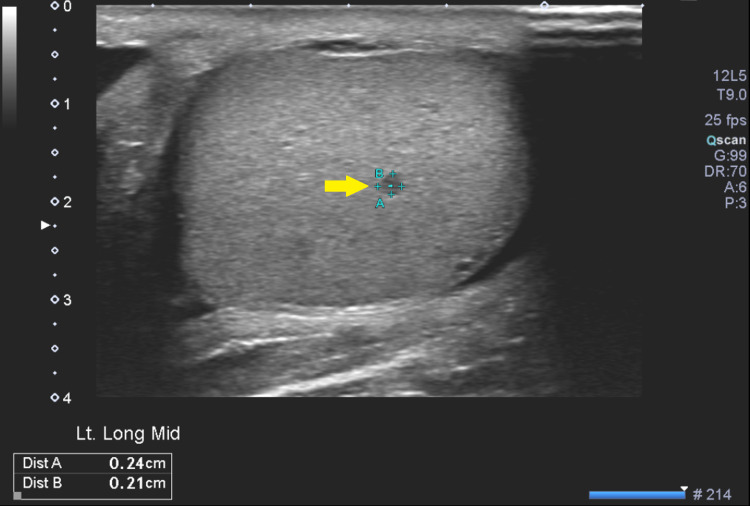
Testicular ultrasound showing typical hypoechoic lesion (with yellow arrow) in the patient.

At follow-up, the patient had a satisfactory response to corticosteroid injections on his skin findings. He had not completed repeat labs for a follow-up on his mild hypercalcemia but was concerned about a non-painful scrotal mass. Ultrasound of the scrotum showed an enlarged epididymis bilaterally with numerous hypoechoic lesions measuring up to 6 mm, along with a left varicocele. Additionally, there were numerous hypoechoic testicular lesions measuring up to 2 mm. The differentials included sarcoidosis or lymphoma. Urology was contacted, and they recommended a biopsy of an easier accessible site rather than the patient’s testicular growths (Figure 1).

The patient followed up within a week, denying any fevers, chills, or night sweats. He had no lymphadenopathy on an exam. He was concerned about right eye erythema and pain. He was urgently sent to ophthalmology due to concern of uveitis. Additionally, blood work and a chest X-ray were obtained. The patient was also sent to dermatology for a skin biopsy, as it was thought that the plaques originally diagnosed and treated as keloids could potentially be skin manifestations of sarcoidosis.

The patient’s angiotensin-converting enzyme (ACE) level was found to be 274 U/L (Reference range: 9-67 units/L), and his IL2R antibodies were 2335 pg/mL (127.3-858.2 pg/mL). Calcium remained elevated at 11.1 (8.4-10.4 mg/dL), 25-OH vitamin D was found to be 7.1 (30-80 ng/mL), and his intact parathyroid hormone (PTH) was 6.7 (14-97 pg/mL). 1,25-dihydroxycholecalciferol was found to be 48.7 pg/mL (19.9-79.3 pg/mL). A chest X-ray was subsequently performed which showed interstitial micronodular disease. The patient’s purified protein derivative (PPD) test was found to be negative. Additionally, ophthalmology diagnosed the patient with uveitis of his right eye, and he was started on appropriate therapy with Pred Forte and Cyclomydril. Sarcoidosis was placed above lymphoma in the differential.

**Figure 2:**
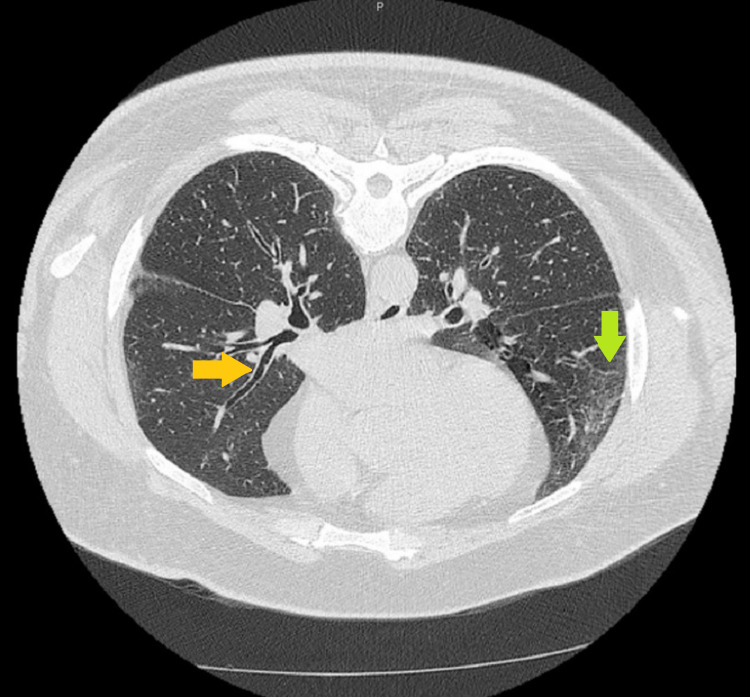
High-resolution CT scan showing a typical pattern of bronchiectasis with a tram track sign is seen in the image (orange arrow). Additionally, micronodules and non-specific ground-glass opacities are seen (green arrow).

A high-resolution CT scan was performed, which showed a nonspecific finding of micronodules and ground-glass opacity, along with bronchiectasis, mediastinal lymphadenopathy, and probable hepatosplenomegaly (Figure 2). Biopsies by the dermatologist on the patient’s left upper arm, right forearm, and right chin showed noncaseating granulomatous dermatitis. The patient was diagnosed with sarcoidosis. The patient’s ultrasound images were reviewed by the urologist, and the patient was reassured that the scrotal ultrasound findings were likely secondary to the recently diagnosed sarcoidosis. The patient did not wish to have any additional biopsies or surgery performed, and as a result, the urologist planned to monitor his testicular masses by ultrasound. Since then, he has additionally been referred to rheumatology and pulmonology for further management.

## Discussion

Genitourinary manifestations of sarcoidosis have been described in case reports in the past. Sixty cases of histologically proven sarcoidosis with GU manifestation had been described prior to 2004. In that small sample size, 48% of the patients were African American, 73% of patients had involvement in the epididymis, and 47% had involvement in the testes [7]. Fewer than 0.2% of all clinically diagnosed cases of sarcoidosis have GU manifestations, but the number is as high as 5% in autopsy studies. Essentially, sarcoid affects the GU system 25 times more frequently than diagnosed [8].

When differentiating sarcoidosis and testicular cancer, it’s paramount to understand that Lactate dehydrogenase (LDH) is not reliable, as sarcoidosis may cause a raised LDH as well. However, it does not cause elevations in alpha-fetoprotein (AFP) or human chorionic gonadotropin (b-HCG) [8]. Clinical findings that increase the likelihood of a benign diagnosis include Afro-Caribbean ethnicity and bilateral testicular lesions [5].

Furthermore, De Cinque et al. suggest using contrast-enhanced ultrasound to differentiate between sarcoidosis and testicular cancers. Although the most accurate method in differentiation remains a biopsy, orchiectomies are often undertaken as it can be misdiagnosed for a neoplasm [9]. On ultrasound, sarcoid shows up as hypoechoic lesions bilaterally, which can be a challenge to differentiate from a malignancy. A clinical picture with signs/symptoms of sarcoidosis or negative tumor markers can assist in forming an accurate diagnosis [10].

There are other concerns with testicular sarcoidosis as well. Kovac et al. report a case of azoospermia from testicular sarcoidosis, resulting in secondary infertility. The case presented with normal follicle-stimulating hormone (FSH) levels and azoospermia after a new-onset difficulty conceiving. A diagnosis of sarcoidosis was made based on CT findings, testicular biopsy, and uveitis. After treatment with corticosteroids, the patient’s sperm concentrations and motility rapidly improved and the couple was able to conceive naturally again [9].

Testicular sarcoidosis continues to pose a multitude of diagnostic challenges and is likely an underdiagnosed entity. To compare, sarcoidosis affects the testes in fewer than 0.2% of all clinically diagnosed cases, while pulmonary manifestations are diagnosed in 90% of cases [8,10]. Management largely depends on the disease severity, the desire for fertility, and symptomatology [11].

## Conclusions

To our knowledge, the amount of literature on testicular sarcoidosis is limited. The diagnosis of testicular sarcoidosis poses unique challenges as well, as it has to be differentiated from testicular cancer, which can result in invasive procedures, such as orchiectomies. This case demonstrates an alternative management strategy that involves a more conservative approach. However, due to the strong association between sarcoidosis and testicular cancer, the patient will have a long-term follow-up with scrotal ultrasounds to ensure that his testicular growths remain stable or resolved.
